# Validation of a shortened version of the Eating Attitude Test (EAT-7) in the Arabic language

**DOI:** 10.1186/s40337-022-00651-5

**Published:** 2022-08-26

**Authors:** Feten Fekih-Romdhane, Sahar Obeid, Diana Malaeb, Rabih Hallit, Souheil Hallit

**Affiliations:** 1 The Tunisian Center of Early Intervention in Psychosis, Department of Psychiatry “Ibn Omrane”, Razi Hospital, 2010 Manouba, Tunisia; 2 Faculty of Medicine of Tunis, Tunis El Manar University, Tunis, Tunisia; 3 Social and Education Sciences Department, School of Arts and Sciences, Lebanese American University, Jbeil, Lebanon; 4 College of Pharmacy, Medical Gulf University, Ajman, United Arab Emirates; 5 School of Pharmacy, Lebanese International University, Beirut, Lebanon; 6 School of Medicine and Medical Sciences, Holy Spirit University of Kaslik, P.O. Box 446, Jounieh, Lebanon; 7 Department of Infectious Disease, Bellevue Medical Center, Mansourieh, Lebanon; 8 Department of Infectious Disease, Notre Dame des Secours University Hospital Center, Street 93, Postal Code 3, Byblos, Lebanon; 9 Psychology Department, College of Humanities, Effat University, Jeddah, 21478 Saudi Arabia; 10 Research Department, Psychiatric Hospital of the Cross, Jal Eddib, Lebanon

**Keywords:** Eating disorders, Eating attitudes test, Psychometric properties, Short version, Arabic language

## Abstract

**Background:**

Eating disorders are quite common around the world, disabling, and potentially lethal; but they remain so far under-captured and subject to substantial delays in treatment. We propose through this study to develop and validate a shortened version of the Eating Attitude Test (EAT) in the Arabic language among non-clinical Arabic-speaking Lebanese participants from the general population.

**Methods:**

1175 participants enrolled in this cross-sectional study, based on an online survey. The Eating Attitude Test-26 items, Drunkorexia Motives and Behaviors Scales and Lebanese Anxiety Scale were used to answer our objectives.

**Results:**

Our results confirmed validity and reliability of the questionnaire. Exploratory Factor Analyses showed that all items converged over a one-factor solution, with an excellent Cronbach’s alpha (> 0.9). Confirmatory Factor Analyses found that the 7-item-version yielded excellent model fit. The instrument also revealed strong evidence of divergent validity, being highly correlated with measures of anxiety and drunkorexia motives and behaviors.

**Discussion:**

Findings provided evidence supporting that the Arabic seven-item one-factor structure of the scale (EAT-7) is valid, reliable, and can be used in clinical practice, preventive interventions and future eating disorders research in Arab settings.

**Supplementary Information:**

The online version contains supplementary material available at 10.1186/s40337-022-00651-5.

## Plain English summary

Eating disorders are quite common around the world, disabling, and potentially lethal; but they remain so far under-captured and subject to substantial delays in treatment. The results of this study showed that a shorter form of the Eating Attitude Test 26 items, the EAT-7, is a valid and reliable instrument that can be used in clinical practice, preventive interventions and future eating disorders research in Arab settings.

**Supplementary Information:**

The online version contains supplementary material available at 10.1186/s40337-022-00651-5.

## Background

Eating disorders (EDs) refer to a wide range of conditions characterized by weight concerns, body image issues and dieting habits [15]. EDs tend to be positively associated with psychiatric morbidity such as anxiety, depression, insomnia [4, 12], alcohol use disorder [25], drunkorexia [5], poor quality of life [44], as well as an elevated risk of suicide [50] and overall mortality [29]. EDs have also been found to cause high healthcare costs and potentially contribute to the global disease burden [14]. Therefore, early detection and intervention of EDs is crucial to improve prognosis, and prevent subsequent morbidity and mortality [42].

While prevalence rates of EDs vary widely according to sample characteristics and screening tools, they were proven to be high in the general population worldwide [45]. For instance, rates in Western countries varied from 0.4% in Spain [18], to 3.9% in Germany, 24.8% in France [53], and 33% in Australia [21]. Rates in non-Western countries appear to be lower but gradually increasing [33]. A more limited research has been undertaken in the Middle East and North African countries [45], with reported prevalence rates of 1–3.3% in Saudi Arabia [1], 24,3% in Tunisia [28], 24.7% in Iran [43], 33.4% in Jordan [39], and 46.2% in Kuwait [13]. The few previous studies done in Lebanon revealed a prevalence of ED in the range of 17–31.4% [2, 8, 22]. These variations across countries and regions are mainly explained by the differences in cultural backgrounds and religious beliefs that considerably affect the prevalence of ED [17, 51].

Eating disorders are even much more common than it was estimated before [45]; and have been for a long time underestimated because of an exclusive focus on bulimia nervosa and anorexia nervosa [45]. However, previous studies revealed that threshold EDs, subclinical EDs, and high-risk behaviors can theoretically span a continuum from asymptomatic eaters to clinically diagnosable EDs [4, 32], with a large variation in symptom’s severity or intensity. Therefore, for a better representation of EDs’ prevalence, a wider specter of eating disorders should be captured using precise, accurate and reliable screening tools. The two-stage methods represent the gold standard for prevalence estimates of EDs [11]. The first stage consists of administering psychometrically valid measures, which help identify individuals at-risk of developing EDs. In the second stage, a structured clinical interview enables to make the diagnosis of a specific EDs.

One of the most commonly used screening tools to assess symptoms and behaviors associated with eating disorders in both clinical and non-clinical settings is the Eating Attitudes Test in its original (EAT-40 [19]) or shortened (EAT-26 [20]) versions. The original version of EAT, developed by Garner et al. [19], comprised 40 self-reported items designed to assess symptoms of anorexia nervosa on a 6-point Likert scale. A short version of 26 items was later developed [20]. The EAT-26 demonstrated excellent psychometric properties, exhibited greater reliability and validity [20], and is suitable in diverse cross-cultural settings. The first developed abbreviated version (EAT-26) is divided into three subscales: dieting (avoidance of fatty foods and preoccupation with thinness), bulimia and food preoccupation, and oral control (self-control over food and social pressure to gain weight) [20]. Though initially designed to differentiate anorexic subjects from normal subjects, the EAT has since been used to identify subjects with different levels and types of eating disturbances [20]. An Arabic version of the EAT-26 has been recently validated by our team [22]. However, due to its length, the EAT-26 can be difficult to complete; and thus complicated to apply practically in the assessment and routine monitoring of EDs in community health programs. In addition, longer scales with more items imply higher cost of public health surveying [7]. Therefore, further abbreviated forms have been developed, including 16-item [35], 13-item [7], 10-item [57], and 8-item [42] scales. These abbreviated versions have been validated in a few languages, including Persian [3] and German [7, 42]; but no Arabic forms, shorter than the EAT-26, exist so far to our knowledge. Expanding and improving the earlier versions of the EAT for Arabic-speaking people around the world would allow for a faster, easier to perform, more convenient, and lower cost screening of EDs in Arab settings.

Therefore, we propose through this study to develop and validate a shortened version of the EAT in the Arabic language. Particularly, we aim to examine its number of factors, internal consistency, and validity in non-clinical Arabic-speaking Lebanese participants from the general population. We expect that the scale will show one factor (H1) and will have a good internal consistency (H2). We also hypothesize that the total score will be positively correlated with anxiety and drunkorexia motives and behaviors (H3).

## Methods

### Study design and procedure

A cross-sectional study, based on an online anonymous survey, was conducted across all the Lebanese governorates, between September and December 2020. The data was collected during lockdowns imposed by the Lebanese government during the COVID-19 pandemic. To avoid person-to-person contact, we used the snowball sampling technique to enroll participants; the survey was shared on social media platforms (WhatsApp, Facebook, LinkedIn). It targeted people aged 18 years old or over (N = 1175). The same procedure was described in the previous publications from this project [5, 34].

### Questionnaire

It was an Arabic anonymous self-administered questionnaire. The estimate time was 10 min. To avoid potential influences, participants were requested to fill it out without help. The first section consisted of sociodemographic variables (age, gender, current weight and height). The Body Mass Index (BMI) was consequently calculated as per the World Health Organization [58]. The Household Crowding Index (HCI), reflecting the socioeconomic status of the family [36], is the ratio of the number of persons living in the house over the number of rooms in it (excluding the kitchen and the bathrooms). The physical activity index is the cross result of the intensity, duration, and frequency of daily activity [56].

The second section included the following measures:

#### Eating attitude test

The EAT, validated in Arabic [22], is used to assess disordered food attitude [19], including twenty-six questions each with six response options, ranging from infrequently/almost never/ never (0) to always (3). The total score is calculated by summing all questions answers and can vary from 0 to 78. A score of 20 or above indicates possible disordered food attitudes [20].

To address our objective in this study, the research team included 10 items from the original scale, which were directly related to food and eating behaviors. We decided to remove all items related to psychological aspect, body shape, etc. since they do not reflect eating attitudes per se.

#### Drunkorexia motives and behaviors scales (DMBS)

The DMBS is a 5-point Likert scale that explores the motives behind engaging in drunkorexia (11 items; e.g. “Because my friends pressure me to restrict my eating”), as well as drunkorexia-type behaviors (12 items; e.g. “eating less at each meal”), which refer to different methods used to restrict calories before an alcohol-drinking episode [55]. The Arabic version of this scale has been used in previous studies [5, 34]. Noting that the DMBS contains 52 items but we chose to use the motives and behaviors subscales in our study.

#### Lebanese anxiety scale (LAS-10)

It is a 10-item instrument, originally constructed in Arabic, and validated among Lebanese individuals [23].

It measures the severity of anxiety symptoms among Lebanese adults [23] and adolescents [37]. In LAS-10, the first seven questions are graded from 1 to 10, and the last three questions are graded from 1 to 4 based on the repetitive manifestation of symptoms. Higher scores indicate higher anxiety level.

### Statistical analysis

The SPSS software v.22 was used for the statistical analysis at first. The sample was randomly divided into two subsamples: sample 1 (N = 597) used to conduct the exploratory factor analysis (EFA) and sample 2 (N = 578) used to conduct the confirmatory factor analysis (CFA). The EFA was run on the ten items we chose from the original EAT-26. The KMO and Bartlett’s test of sphericity values ensured model’s adequacy. The SPSS AMOS v.24 was used to conduct the CFA; the root mean square error of approximation (RMSEA) statistic, the Tucker Lewis Index (TLI) and the comparative fit index (CFI) were used to evaluate the goodness-of-fit of the model as these are the most commonly used indices. Schermelleh-Engel et al. [47] recommended RMSEA values ≤ 0.06, with TLI and CFI values ≥ 0.95 for an excellent-fit model.

To examine gender invariance of the EAT-7 scores, we conducted multi-group CFA [9] using the total sample. Measurement invariance was assessed at the configural, metric, and scalar levels [54]. Configural invariance implies that the latent EAT-7 variable and the pattern of loadings of the latent variable(s) on indicators are similar across gender (i.e., the unconstrained latent model should fit the data well in both groups). Metric invariance implies that the magnitude of the loadings is similar across gender; this is tested by comparing two nested models consisting of a baseline model and an invariance model. Lastly, scalar invariance implies that both the item loadings and item intercepts are similar across gender and is examined using the same nested-model comparison strategy as with metric invariance [9]. Following the recommendations of Cheung and Rensvold [10] and Chen [9], we accepted ΔCFI ≤ 0.010 and ΔRMSEA ≤ 0.015 as evidence of invariance. The computed EAT-7 score was normally distributed; to test the divergent validity, the Pearson correlation test was used to correlate it with other continuous variables. The independent sample t test was used to check for a difference between genders in terms of EAT. *P* < 0.05 was considered statistically significant.

## Results

The mean age in both samples was around 25 years, with the majority composed of females. Other details about the two samples can be found in Table 1.

**Table 1:** Sociodemographic characteristics of the samples

Variable	Sample 1 (N = 597)	Sample 2 (N = 578)
Age (in years)	24.87 ± 8.42	25.43 ± 8.64
Physical activity index	25.31 ± 19.29	26.27 ± 19.89
Household crowding index (person/room)	1.13 ± 0.66	1.10 ± 0.58
Body mass index (kg/m^2^)	24.28 ± 5.06	24.00 ± 4.78
Gender		
Male	175 (30.3%)	178 (29.8%)
Female	403 (69.7%)	419 (70.2%)

### Exploratory factor analyses (Sample 1)

All items selected in the three models were extracted and converged over a one-factor solution, with items having loading factors > 0.4 and communalities > 0.3. The Cronbach’s alpha values for the three tested models were excellent (> 0.9) (Table 2).

**Table 2:** Factor analyses of the three models of the Eating Attitude Test

	Model 1		Model 3		Model 3	
	Loading	Communality	Loading	Communality	Loading	Communality
EAT 2	0.792	0.627	0.820	0.672	0.795	0.631
EAT 3	0.763	0.583	0.761	0.579		
EAT 4	0.716	0.513				
EAT 6	0.795	0.631	0.794	0.630	0.814	0.663
EAT 7	0.837	0.701	0.854	0.730	0.849	0.721
EAT 15	0.719	0.516	0.729	0.531	0.727	0.528
EAT 16	0.838	0.701	0.869	0.755	0.876	0.767
EAT 17	0.851	0.724	0.841	0.707	0.858	0.737
EAT 22	0.828	0.685	0.846	0.715	0.828	0.686
EAT 23	0.819	0.671				
Variance explained	63.54		66.47		67.62	
Cronbach’s alpha	0.94		0.93		0.92	

Model 1: EAT including 10 items; KMO = 0.929; Bartlett’s test of sphericity *P* < 0.001; Model 2: EAT including 8 items; KMO = 0.932; Bartlett’s test of sphericity *P* < 0.001; Model 3: EAT including 7 items; KMO = 0.919; Bartlett’s test of sphericity *P* < 0.001

### Confirmatory factor analyses (Sample 2)

**Fig. 1:**
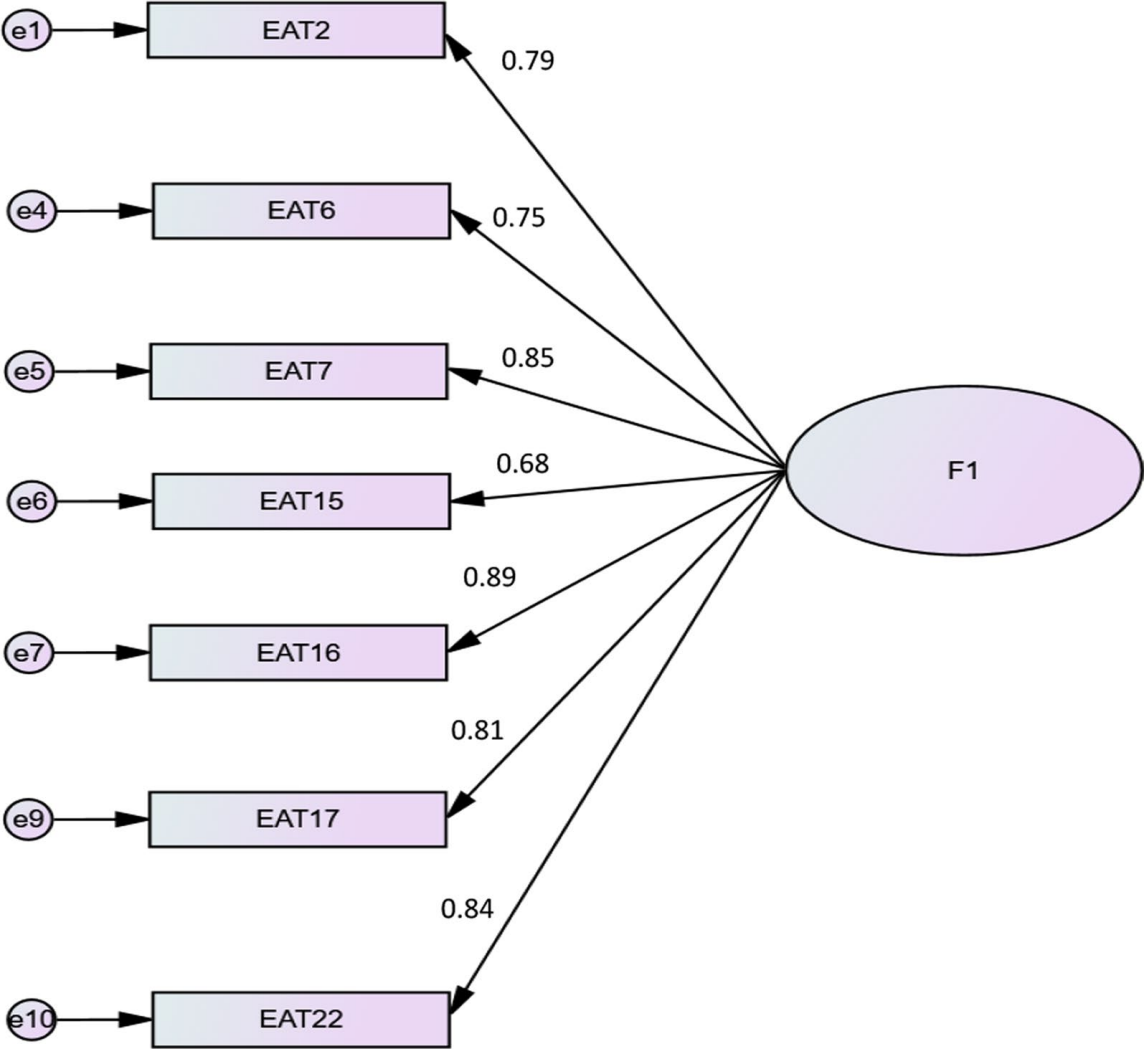
Standardized factor loadings of the short form of the Eating Attitude Test (EAT-7)

We first selected 10 items. The CFA fit indices were not good, with high modification indices between items 3 and 4 and between 17 and 23 (Table 3, Model 1). We conducted a second CFA after removing items 4 and 23. The CFA fit indices significantly improved but some of them were still below the set cutoff values, with high modification indices between items 2 and 3 and between items 3 and 16 (Table 3, Model 2). Therefore, we conducted a third CFA after removing item 3 and obtained excellent fit indices, with acceptable modification indices (Table 3, Model 3). The final form of the EAT-7 can be found as Additional file 1. The standardized factor loadings of the short form of the Eating Attitude Test (EAT-7) are summarized in Fig. 1.

**Table 3:** Fit indices of the three tested confirmatory factor analysis models of the Eating Attitude Test items

	χ^2^_(df)_	*P*	TLI	CFI	RMSEA	90% CI
Model 1	396.29_(35)_	< 0.001	0.90	0.92	0.13	0.122, 0.146
Model 2	130.71_(20)_	< 0.001	0.95	0.97	0.10	0.082, 0.114
Model 3	68.69_(14)_	< 0.001	0.97	0.98	0.08	0.063, 0.102

Model 1: EAT including 10 items; Model 2: EAT including 8 items; EAT including 7 items

### Divergent validity (Sample 2)

Higher EAT-7 scores were significantly associated with higher anxiety (r = 0.11; *P* = 0.009), higher drunkorexia motives (r = 0.40; *P* < 0.001) and higher drunkorexia behaviors (r = 0.46; *P* < 0.001).

### Measurement invariance

As reported in Table 4, all indices suggested that configural, metric, and scalar invariance was supported across gender.

**Table 4:** Measurement Invariance Across Gender

Model	χ^2^	*df*	CFI	RMSEA	Model Comparison	Δχ^2^	ΔCFI	ΔRMSEA	Δ*df*	*P*
Configural	116.80	28	0.984	0.052						
Metric	135.10	34	0.982	0.050	Configural vs metric	18.30	0.002	0.002	6	0.530
Scalar	119.43	34	0.984	0.046	Metric vs scalar	15.67	0.002	0.004	6	0.025

*CFI* comparative fit index, *RMSEA* Steiger-Lind root mean square error of approximation

Given these results, we computed an independent-samples *t*-test to examine gender differences in EAT-7 scores. The results showed that no significant difference between women (*M* = 5.73, *SD* = 6.57) and men (*M* = 6.07, *SD* = 7.01) in terms of eating disorders, *t*(1173) = 0.78, *P* = 0.433.

In addition, higher EAT-7 scores were significantly associated with lower household crowding index (r = -0.13; *P* < 0.001), but not age (r = 0.02; *P* = 0.594) or BMI (r = 0.03; *P* = 0.353).

## Discussion

EDs are quite common around the world, disabling, and potentially lethal, but remain so far under-captured and subject to substantial delays in treatment [24]. Screening for EDs via valid and reliable scales is highly important for clinical and preventive practice, as well as for research. Short scales requiring less effort and time from respondents are recommended, provided they have “enough items to keep the alpha within the acceptable range” [38]. We believe that a validation of the Arabic shortened version of the EAT is required to improve our understanding of the usefulness of the EAT in Arab clinical and research settings. Our investigation of the psychometric characteristics of the Arabic EAT-7 showed a robust, adequate, and reliable unidimensional factor structure. The instrument also revealed strong evidence of divergent validity, being highly correlated with measures of anxiety and drunkorexia motives and behaviors. In sum, we suggest that the Arabic EAT-7 is highly convenient and suitable for use via self-report to screen for EDs risk in Arabic-speaking non-clinical populations.

We evaluated the psychometric proprieties of the EAT-7 through exploratory and confirmatory factor analyses, internal reliability consistency (α > 0.9), and discriminant validity; and the data provided evidence for the reliability and validity of the seven-item one-factor structure of the scale for a non-clinical adult community sample in Lebanon. For short scales with a limited number of items, a Cronbach’s alpha value ranging from 0.8 to 0.9 is considered “ideal” [38], which highlights the strong reliability of the EAT-7. Our investigation supported a unidimensional construct of the EAT-7, which represents its major strength since it allows for an interpretation of a (total) test score [52]. Because of its same underlying construct, the total EAT-7 score is accurate and precise [52]. For clinical practice, the one-factor structure of the EAT-7 is important, enabling clinicians to make valid inferences about its score and the underlying construct that provided this score [52]. The fact that one construct is assessed at a time is potentially relevant; especially when aiming to compare scores between different respondents, or for the same respondent over time [52].

Furthermore, we explored the relationship between the EAT-7 and anxiety and drunkorexia motives and behaviors. Our analyses revealed significant, strong and positive correlations between these entities; which aligns with previous findings. Indeed, the accumulated evidence point to a significant overlap between EDs and anxiety disorders; with shared risk factors, clinical symptoms, temperamental profiles, and comorbidities [46]. Similar patterns of association were found between EDs and drunkorexia [16, 27, 33, 49]. Drunkorexia refers to feeding problems, coupled with excessive alcohol consumption and/or physical activity [41]. Drunkorexia has been found to include an overlap between alcohol use and inappropriate eating habits [30, 31]. At the same time, EDs were demonstrated to be significantly correlated with alcohol use [6], with both entities being potential predictors of engaging in drunkorexic behaviors [26]. Confirming the relationship of the EAT-7 with anxiety levels and drunkorexia tendencies is foundational to draw conclusions about the scale score precision and integrity. In our case, the positive correlations of the EAT-7 to the LAS-10 and DMBS scores helped provide a better understanding of how individuals who engage in EDs exhibit symptoms of anxiety, and tend to get also involved in drunkorexic motives and behaviors. By gaining more knowledge about the links of EAT-7 to LAS-10, and EAT-7 to DMBS, both clinicians and researchers can strengthen and advance their interpretation the EAT-7’s limitations and contributions as compared to other ED measures.

### Limitations and research implications

A number of strengths should be highlighted. An adequate sample size was used and individuals of both genders participated in the study. In addition, validating and examining the psychometric properties of a shortened version of the EAT in an Arab-speaking society and a developing country of the Middle East region may represent a significant advancement to the field. Such research emerging from an under-explored cultural environment, for a culturally dependent topic, might help with the external validity of the instrument.

At the same time, some limitations should be acknowledged and addressed in future research. The main limitation lies to the exclusive inclusion of a non-clinical sample and the fact that it was collected via the snowball technique, precluding any generalization of our conclusions to the EDs patients’ population. In order to be able to make assumptions regarding patients with EDs, we suggest that the psychometric properties of the EAT-7 should be further investigated in clinical populations using control groups. Another limitation consists of the inclusion of participants from one Arab country and culture, Lebanon. However, while the different Arab countries have several social, religious and cultural similarities [40, 48], diversities do also exist; emphasizing the need for future cross-cultural validations of the EAT-7 across the various local contexts of each country.

## Conclusion

This study sought to examine the psychometric properties of the EAT-7; and provided evidence that it could be used to screen for ED research as a valid and reliable measure with its seven items and one factor structure. Our findings provide support that the scale is convenient for use in clinical practice, preventive interventions and future ED research in Arab settings. More cross-cultural validations of the EAT-7 across the various cultural and religious contexts of each Arab country are still required.

## Supplementary Information

**Additional file 1**. The final form of the EAT-7.

## Data Availability

The authors do not have the right to share any data information as per the ethics committee rules and regulations. Data is available upon a valid and reasonable request from the corresponding author (S.H.).
