# Supplementary material for: Validation of a shortened version of the Eating Attitude Test (EAT-7) in the Arabic language
Source: J Eat Disord. 2022 Aug 26;10:127. doi: 10.1186/s40337-022-00651-5 (PMC9412802; doi:10.1186/s40337-022-00651-5)
Supplement: Supplementary file 1 — Additional file 1. The final form of the EAT-7. [file 40337_2022_651_MOESM1_ESM.docx]

| **اختبار المواقف من الأكل (EAT-7)** | | | | | | |
| --- | --- | --- | --- | --- | --- | --- |
| فيما يلي قائمة من العبارات التي تصف المواقف من الأكل. يرجى إختيار العبارة التي تقدّم أفضل وصف لحالتك علماً أنه لا توجد إجابات صحيحة أو خاطئة. | | | | | | |
| **الأسئلة** | **دائماً**  **(3)** | **عادةً**  **(2)** | **غالباً**  **(1)** | **أحياناً**  **(0)** | **نادراً**  **(0)** | **أبداً**  **(0)** |
| 1. أتجنّب الاكل عندما أكون جائع |  |  |  |  |  |  |
| 1. أدرك محتوى السعرات الحرارية في الأطعمة التى أتناولها |  |  |  |  |  |  |
| 1. أتجنب بشكل خاص الأطعمة التى تحتوي الكثير من الكربوهيدرات (مثلاً: خبز، أرز، بطاطا الخ...) |  |  |  |  |  |  |
| 1. أستغرق وقت أطول من الآخرين لتناول وجبة الأكل |  |  |  |  |  |  |
| 1. أتجنب الاطعمة التي تحتوي على السكر |  |  |  |  |  |  |
| 1. أتناول أطعمة للحمية |  |  |  |  |  |  |
| 1. أشعر بالانزعاج بعد اكل الحلويات |  |  |  |  |  |  |
